# Comprehensive phylogeny of Pieridae butterflies reveals strong correlation between diversification and temperature

**DOI:** 10.1016/j.isci.2024.109336

**Published:** 2024-02-28

**Authors:** Ana Paula S. Carvalho, Hannah L. Owens, Ryan A. St Laurent, Chandra Earl, Kelly M. Dexter, Rebeccah L. Messcher, Keith R. Willmott, Kwaku Aduse-Poku, Steve C. Collins, Nicholas T. Homziak, Sugihiko Hoshizaki, Yu-Feng Hsu, Athulya G. Kizhakke, Krushnamegh Kunte, Dino J. Martins, Nicolás O. Mega, Sadaharu Morinaka, Djunijanti Peggie, Helena P. Romanowski, Szabolcs Sáfián, Roger Vila, Houshuai Wang, Michael F. Braby, Marianne Espeland, Jesse W. Breinholt, Naomi E. Pierce, Akito Y. Kawahara, David J. Lohman

**Affiliations:** 1 McGuire Center for Lepidoptera and Biodiversity, Florida Museum of Natural History, Gainesville, FL, USA; 2 Center for Global Mountain Biodiversity, Globe Institute, University of Copenhagen, Copenhagen, Denmark; 3 Center for Macroecology, Evolution, and Climate, Globe Institute, University of Copenhagen, Copenhagen, Denmark; 4 Florida Museum of Natural History, University of Florida, Gainesville, FL, USA; 5 Department of Entomology, Smithsonian Institution, National Museum of Natural History, Washington, DC, USA; 6 Department of Natural Sciences, Bernice Pauahi Bishop Museum, Honolulu, HI, USA; 7 Biology Department, Howard University, Washington, DC, USA; 8 African Butterfly Research Institute, Karen, Nairobi, Kenya; 9 Department of Entomology and Nematology, University of Florida, Gainesville, FL, USA; 10 Department of Agricultural and Environmental Biology, Graduate School of Agricultural and Life Sciences, The University of Tokyo, Bunkyo-ku, Tokyo, Japan; 11 Department of Life Science, National Taiwan Normal University, Taipei, Taiwan, R.O.C; 12 National Centre for Biological Sciences, Tata Institute of Fundamental Research, GKVK Campus, Bengaluru, India; 13 Turkana Basin Institute, Stony Brook University, Stony Brook, NY, USA; 14 Insect Committee of Nature Kenya, The East Africa Natural History Society, Nairobi, Kenya; 15 Programa de Pós-Graduação em Biologia Animal, Universidade Federal do Rio Grande do Sul, Porto Alegre, RS, Brazil; 16 Saitama Study Center, The Open University of Japan, Omiya-ku, Saitama City, Japan; 17 Museum Zoologi Bogor, Research Center for Biosystematics and Evolution, Research Organization for Life Sciences and Environment, National Research and Innovation Agency, Cibinong, Bogor, Indonesia; 18 Laboratório de Ecologia de Insetos, Departamento de Zoologia, Instituto de Biociências, Universidade Federal do Rio Grande do Sul, Porto Alegre, RS, Brazil; 19 Institute of Silviculture and Forest Protection, University of Sopron, Sopron, Hungary; 20 Institut de Biologia Evolutiva (CSIC-Univ. Pompeu Fabra), Barcelona, Spain; 21 Department of Entomology, College of Plant Protection, South China Agricultural University, Guangzhou, China; 22 Division of Ecology and Evolution, Research School of Biology, The Australian National University, Acton, ACT, Australia; 23 Australian National Insect Collection, National Research Collections Australia, Canberra, ACT, Australia; 24 Leibniz Institute for the Analysis of Biodiversity Change, Museum Koenig, Bonn, Germany; 25 Intermountain Healthcare, Intermountain Precision Genomics, St. George, UT, USA; 26 Department of Organismic and Evolutionary Biology, Museum of Comparative Zoology, Harvard University, Cambridge, MA, USA; 27 Department of Biology, University of Florida, Gainesville, FL, USA; 28 Department of Biology, City University of New York, New York, NY, USA; 29 PhD Program in Biology, Graduate Center, City University of New York, New York, NY, USA; 30 Entomology Section, National Museum of Natural History, Manila, Philippines

**Keywords:** Entomology, Evolutionary biology, Phylogeny

## Abstract

Temperature is thought to be a key factor influencing global species richness patterns. We investigate the link between temperature and diversification in the butterfly family Pieridae by combining next generation DNA sequences and published molecular data with fine-grained distribution data. We sampled nearly 600 pierid butterfly species to infer the most comprehensive molecular phylogeny of the family and curated a distribution dataset of more than 800,000 occurrences. We found strong evidence that species in environments with more stable daily temperatures or cooler maximum temperatures in the warm seasons have higher speciation rates. Furthermore, speciation and extinction rates decreased in tandem with global temperatures through geological time, resulting in a constant net diversification.

## Highlights

• The most comprehensive phylogeny of Pieridae to date, with 593 pierid species
• Mild warm seasons are associated with higher speciation rates
• Habitats with high daily temperatures host species with lower speciation rates
• Diversification decreased in tandem with global temperatures through time

## Graphical abstract

**Figure:**
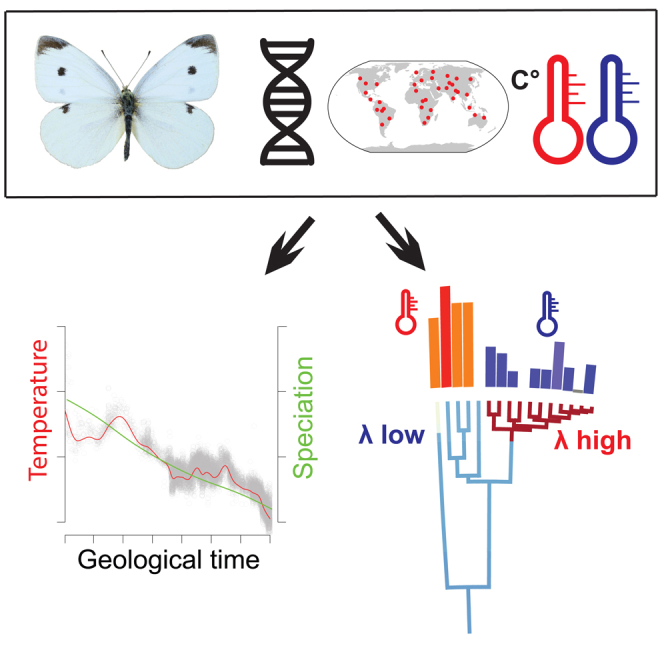

## Introduction

Understanding drivers of species diversity across Earth is a central pursuit of ecology and evolutionary biology.^1^ While multiple biotic and abiotic factors can influence speciation and extinction,^2^^,^^3^^,^^4^^,^^5^ temperature is thought to be a key variable impacting spatial diversity patterns.^6^^,^^7^^,^^8^^,^^9^ Diversity is often greater in warmer environments due to higher evolutionary rates,^2^ as these species tend to have higher mutation rates, faster metabolism, and shorter generation times, all of which are directly associated with increased rates of evolution.^2^^,^^4^^,^^10^^,^^11^^,^^12^ Due to the lack of pronounced temperature variation in species-rich areas (e.g., some tropical regions), authors also hypothesize that temperature stability could affect diversification.^13^ This relationship is based on the premise that climatic stability favors the evolution of specialized adaptations (climatic stability hypothesis^13^). The inherent temperature stability of tropical climates compared to temperate ones is therefore thought to reduce extinction rates.

To examine whether temperature is correlated with diversification, it is necessary to study widely distributed clades of species inhabiting diverse biomes that have sufficiently fine-grained spatial data. Additionally, a well-supported, dated phylogenetic tree is necessary to account for relationships among species when evaluating patterns of diversity. Although many studies have assessed the correlation between ambient temperature and diversity, these studies have primarily focused on vertebrates and plants.^3^^,^^14^^,^^15^^,^^16^^,^^17^^,^^18^^,^^19^^,^^20^^,^^21^^,^^22^ Considerably less attention has been directed to other, substantially more diverse groups like insects, which—as ectotherms—have life histories more likely to be intrinsically connected to temperature.^23^ Butterflies are among the best documented insect groups, with nearly 34.5 million locality records in the Global Biodiversity Information Facility (GBIF, as of September 2022).

Studies of butterfly diversification have largely focused on the coevolution with their host plants.^24^^,^^25^ Prior investigations have found strong evidence for reciprocal adaptations between Pierinae butterflies and their host plants as predicted by an escape-and-radiate coevolutionary scenario.^26^^,^^27^ However, an extensive study examining evolutionary patterns of host use across all butterflies found weak evidence for coevolution in many clades.^25^ Host plants are just one factor that might affect insect diversification rates; less attention has been paid to the role of climate.

Butterfly species richness is generally much higher in equatorial regions than temperate regions,^28^ and is also correlated with ambient temperature.^6^^,^^29^ As with other ectotherms, temperature strongly influences insect larval development and diapause.^30^ Ambient temperature and sunlight have direct effects on behaviors, such as basking, and indirect effects on life history traits, including phenology and voltinism.^31^^,^^32^ Consequently, extreme or highly variable temperatures can negatively impact fitness.^33^ Temperature-mediated diversification is another important context in which to study diversification dynamics in ectothermic organisms such as butterflies.

**Figure 1:**
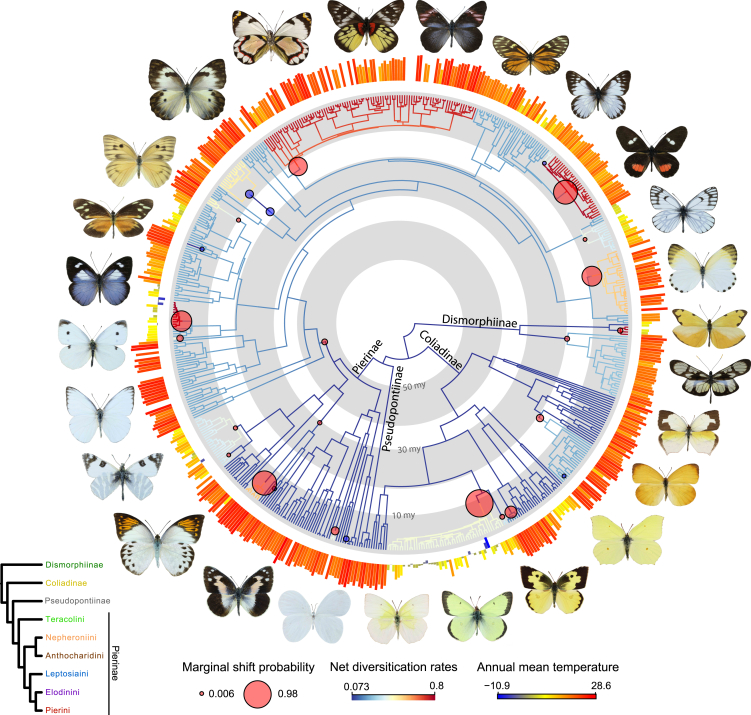
Evolutionary relationships and diversification patterns of Pieridae Time-calibrated tree of 593 species. Branches with significant net diversification rate shifts as estimated by BAMM are indicated with red and blue circles. Annual mean temperature (BIO1, WorldClim) for each species is indicated with colored bars in the outer ring. Butterfly images are not to scale. Bottom left inset shows a cladogram of the subfamily and tribal relationships in this figure.

The family Pieridae, sometimes called the white and yellow butterflies, comprises at least 1,159 species in 86 genera distributed across the globe.^34^ Most of their diversity is concentrated in the tropics,^35^ but Pieridae is known for having species adapted to extreme cold or dry conditions, such as high montane habitats,^36^ the Arctic^37^ and deserts.^38^ The family is sensitive to ambient temperature, and morphological adaptations for regulating temperature have been identified in several lineages.^39^^,^^40^^,^^41^^,^^42^ Pierid monophyly is well established,^43^^,^^44^ but relationships among subfamilies and tribes remain contentious (see Braby M.F. and Pierce N.E., Braby M.F. et al., and Warren-Gash H. et al^45^^,^^46^^,^^47^ for clade- or region-specific studies) (Figure 1), and current pierid phylogenies are neither sufficiently robust nor comprehensive enough to address macroevolutionary questions. A robust phylogeny of Pieridae will not only resolve its evolutionary relationships but also help answer questions regarding the role of thermal ecology in insect macroevolution. Here, we reconstruct a comprehensive species-level dated tree of Pieridae with 593 species from 84 genera and infer their diversification patterns, wherever possible accounting for incomplete sampling. We combine our phylogenetic reconstruction with climate data extracted using distribution records to examine how speciation and extinction are correlated with paleotemperatures and how present-day (1) warmer climates (annual mean temperature), (2) seasonality and daily temperature variability (temperature annual range and annual mean diurnal range), and (3) extreme temperatures (maximum temperature of the warmest month and minimum temperature in the coldest month) impact pierid diversity. We evaluated three related hypotheses, phrased here as questions: (1) Are temperature stability and warmer climates associated with higher pierid diversity as predicted by the climatic stability hypothesis?; (2) How does climate variability over time and space impact diversification dynamics?; and (3) What aspects of climate are most strongly associated with diversification rate shifts?

## Results

### Pieridae phylogeny

**Figure 2:**
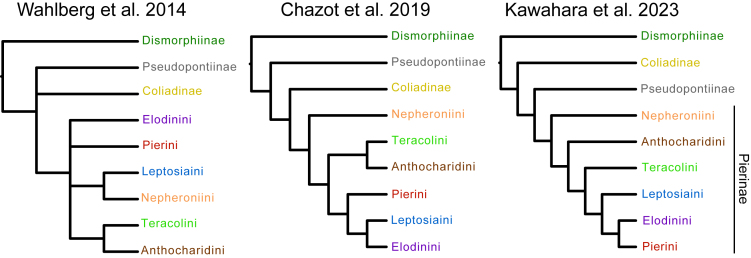
Summary of the inferred relationships among higher-level Pieridae taxa in recent molecular phylogenetic studies.

We inferred the most taxonomically comprehensive phylogeny of Pieridae to date (Figures 1 and S1), including genetic data for 593 pierid species and 15 outgroups. Eighty-four of the 86 Pieridae genera recognized by Lamas^34^ are included; the two missing genera, *Calopieris* and *Piercolias* are rarely collected, and the validity of the latter has been recently questioned.^48^ Subfamilial relationships were strongly supported (UFBoot and SH-aLRT >95%) in general, but support for the position of Pseudopontiinae, which we recovered as the sister group to Pierinae, was low (UFBoot = 75.2, SH-aLRT = 77). This corroborates the results of Kawahara et al.^25^ and Espeland et al.^49^ based on 391 loci, but not those of Chazot et al.^50^ which used nine loci (Figure 2). Recently, Zhang et al.^48^ used phylogenomic analyses to validate previous studies indicating the non-monophyly of some taxa. They suggested that *Catasticta* should be a junior synonym of *Archonias* and that *Eurema* is paraphyletic, among other issues. Regarding *Catasticta* and *Archonias*, for example, phylogenetic, morphological, and natural history traits had already suggested that these genera are closely related to each other.^46^^,^^51^^,^^52^ However, sampling in Zhang et al.^48^ was limited in some clades, and our study highlights additional issues. Namely, *Euchloe* is paraphyletic, *Rhabdodryas* is nested within *Phoebis*, *Glennia* is nested in *Ganyra*, and *Eurema* is still not monophyletic even after proposed changes by Zhang et al*.*^48^

Divergence time estimates varied depending on the calibration scheme, with calibrations taken from Kawahara et al.^53^ resulting in younger dates (Table S1). The crown age of Pieridae was between 69.6 and 64.5 Ma with a stem age between 83.5 and 76.1 Ma. Subfamily crown ages were estimated to be 35.0–32.7 Ma for Dismorphiinae; 49.8–44.2 Ma for Coliadinae; and 49.9–48.9 Ma for Pierinae (Table S1). The five Afrotropical Pseudopontiinae species are on a long branch with extant taxa diversifying only 5.1–3.8 Ma (Table S1).

### Diversification patterns of Pieridae

Pierid lineages have steadily increased since the family’s origin around 70 Ma, according to the deterministic lineage through time plot (dLTT) (Figure S2). In contrast, fitted pulled speciation rates (PSR) gradually decreased from the stem lineage until 40 Ma, when rates started to increase, followed by two peaks between 20 Ma and the present (Figure S3).

The BAMM (Bayesian Analysis of Macroevolutionary Mixtures) estimation of net diversification rates identified five clades with increased diversification rates: *Colias*; a subclade of *Catasticta* (+*Archonias*); *Delias*; and clades within *Pieris* and *Mylothris* (Figures 1 and S4). These patterns were consistent with our RevBayes analysis (Figure S5). The distinct shift configuration of BAMM suggests a high (>0.75) marginal probability of shifts for *Colias*, *Pareronia*, and a subclade of *Catasticta* (+*Archonias*) (Figure S4).

### Pieridae diversity and temperature through time

An ancestral state reconstruction of annual mean temperature indicates that the ancestor of Pieridae was likely found in warm climates. Switches to colder, temperate climates occurred multiple times, such as in the genera *Colias* and *Pieris* (Figure S6), and the reconstruction of minimum temperature in the coldest month shows pronounced habitat shifts to colder temperatures in these genera (Figure S7). There was a clear expansion toward habitats with greater annual temperature ranges (seasonality) in *Aporia*, *Colias*, and *Pieris* (Figure S8). Reconstructions of mean diurnal range (Figure S9) and maximum temperature in the warmest month (Figure S10) did not show pronounced variation across the tree.

The best-fit model explaining Pieridae diversification dynamics was a positive exponential relationship for both speciation and extinction rates with (paleo)temperature (BEnv.VarDEnv.Var_EXPO) (Table S2). Global temperature generally decreased during the past ∼40 My during which most of the Pieridae crown group evolved. Thus, the exponential relationships of speciation and extinction rates with paleoclimate mean these rates also decreased (slowed) with time toward the present. The best model fit for the largest Pieridae subfamily, Pierinae, describes a pattern in which speciation rate was constant but extinction rate decreased with paleotemperature (BCSTDEnv.Var_EXPO), meaning that net diversification remained positive in the group.

### Present day temperature and Pieridae diversity

Our phylogenetic generalized least squares (PGLS) analyses found significant relationships between current temperature and speciation/extinction for two variables: (1) minimum temperature of the coldest month (positive for speciation, negative for extinction) and (2) annual temperature range (negative for speciation, positive for extinction). We also found a significant negative relationship between speciation and mean diurnal range (Figure S11). The annual mean temperature and maximum temperature of the warmest month was not significantly related to speciation or extinction.

When QuaSSE (Quantitative State Speciation and Extinction) was used to fit different likelihood functions of speciation on the tree for different temperature-related variables, the only significant (ΔAIC >2) variables were (1) mean diurnal range and (2) maximum temperature in the warmest month (Table S3). We found a negative sigmoidal relationship with drift between speciation and these two variables (https://doi.org/10.5061/dryad.c59zw3rbh). This means that speciation rates are higher in habitats with low diurnal temperature ranges and with mild summer temperatures, but there is a steep decline in speciation rates when the diurnal range exceeds 11°C and, separately, when summer temperatures are higher than 31°C.

PGLS and QuaSSE both indicated a negative relationship between speciation and mean diurnal temperature range (Table S3). The PGLS analysis of maximum temperature of the warmest month was not significant, but QuaSSE suggests a negative correlation as the best fit for this variable, indicating that extremely high temperatures negatively affect speciation rates. The direction of correlations between speciation and minimum temperature of the coldest month and temperature annual range differed between the two methods. However, PGLS p values were significant while the QuaSSE ΔAIC was less than 2. These results suggest that milder temperatures in the coldest month and less variable temperature throughout the year are correlated with higher speciation.

The discrepancies between PGLS and QuaSSE are likely an algorithmic artifact. The PGLS used tip rate estimations extracted from BAMM, while QuaSSE evaluated correlations using its own lambda (speciation) and mu (extinction) estimates. Additionally, QuaSSE fits the data to five different models while PGLS is limited to a linear regression, which may not be the best model for the data.

## Discussion

### Temperature stability and warmer climates are associated with higher diversification over time

Paleotemperatures partially explain current pierid diversification patterns. Throughout their 70 My evolutionary history, the diversification rate of Pieridae is positively associated with warmer temperatures. The MRCA of the family likely inhabited warm climates, and speciation and extinction rates remained positive but have slowed along with global cooling that began in the late Eocene. Thus, higher diversification rates are associated with warmer temperatures through time.

Interestingly, our investigations of diversification dynamics in the most species-rich subfamily, Pierinae, are different from those of the family as a whole. Net diversification has increased since the Cenozoic as a result of decreasing extinction rates combined with constant speciation. Future research could further clarify these dynamics by examining smaller clades within Pieridae but with complete or nearly complete species-level sampling.

### Temperate climates are associated with higher speciation across space

From their likely tropical origins, there have been numerous shifts from tropical to temperate areas with greater temperature variability throughout the year, and several of these shifts are associated with increased diversification, notably in the genera *Colias* and *Pieris*. *Delias*, the most species-rich genus of butterflies, is often associated with equatorial habitats, but many species are found in environments with cooler temperatures (Figure S6) including tropical and subtropical mountains.

### High variability in daily temperatures is associated with decreased diversification

We employed several analyses to identify the specific climatic factors associated with diversification rate shifts. Different analyses gave conflicting results about which aspects of temperature play an outsized role in determining extant diversity. PGLS analyses concluded that (1) diversification increases in cold areas (increased speciation and decreased extinction associated with lowest temperature in the coldest month); (2) diversification declines in areas with large temperature fluctuations throughout the year (lower speciation and increased extinction associated with annual temperature range); and (3) speciation declines in areas with large temperature fluctuation throughout the 24 h day (mean diurnal range). Annual mean temperature and hottest temperature of the warmest month were not significant in these analyses. On the other hand, the QuaSSE analysis identified this latter variable as significant, along with mean diurnal range. The QuaSSE analysis indicated that speciation rates are higher in habitats with low daily temperature variation (<11°C) and with cool summer temperatures that rarely exceed 31°C. We therefore tentatively conclude that large daily fluctuations in temperature—a feature of temperate and montane habitats—are associated with increased speciation, a conclusion on which both analyses agree.

Seasonality influences species’ distributions^54^ and is thought to be correlated with butterfly species richness.^55^ In general, biodiversity is greater in stable, tropical environments, and these areas are often characterized by high temperatures that are generally more constant throughout the year.^56^ In warm seasons, sunlight and ambient temperature are the main factors influencing butterfly activity, and if conditions are not ideal, butterflies will shift the timing and length of their activity.^57^ The diversification of white and yellow butterflies is higher in these environments with stable circadian temperatures. The climatic stability hypothesis^13^ predicts that the tropics are especially diverse in part because the climate does not fluctuate much throughout the day or year. We found no support for this hypothesis in Pieridae. However, one corollary of this idea was supported: large daily temperature fluctuations (lack of stability) are associated with decreased diversification (specifically, decreased speciation).

Although warmer environments are often linked to life history traits that accelerate speciation,^10^^,^^11^^,^^12^ we found that speciation rates are elevated in temperate environments. Butterflies have an optimal internal temperature range for survival but can tolerate greater environmental ranges because they have some ability to control their thermal physiology.^31^^,^^42^^,^^58^ Nonetheless, anything below or above the tolerable range can be lethal.^31^ The optimal temperature range usually trends toward warmer temperatures, and most species are active during warm seasons. In some cases, the warmest month is the only time of the year when butterflies can reproduce. This is especially true for Arctic and alpine species. However, extremely warm temperatures can reduce activity in some species.^57^ Recognizing these trends is crucial in light of the current, unprecedentedly rapid increase in global temperatures during the Anthropocene, particularly our evidence that speciation rates decrease with increasing temperature.

Franzén et al.^57^ found that the lifespan of the Palearctic papilionid *Parnassius apollo* is negatively affected by increased median temperatures and that the lycaenid *Phengaris arion* lives longest at an optimal temperature of *ca.* 27°C, suggesting stabilizing selection on temperature. In general, if temperatures are extreme, behavior and morphology/wing coloration will adapt to regulate body temperature.^40^^,^^59^^,^^60^^,^^61^ Butterflies use different strategies and mechanisms to maintain body temperature, which can disrupt their regular behavior.^42^ They may, for example, find a shaded area to hide,^31^ which then prevents them from feeding and finding mates.

There is a uniform distribution of taxa in warm regions with mild winters across the Pieridae phylogeny (Figures S6 and S7), which is unsurprising considering Pieridae are generally most diverse in warm climates (as in other butterfly families^62^). However, there are multiple independent origins of cold-adapted species (e.g., *Baltia* spp. and *Reliquia santamarta*) including clades that are sister to taxa inhabiting warm or mild climates (such as the clade that includes *Tatochila*, *Hyposchila*, *Theochila*, *Pierphulia*, *Phulia*, and *Infraphulia*). Some of these shifts are associated with adaptations to habitats at higher elevations, as shown by the convergence of morphology among groups that inhabit similarly cold, high-elevation habitats around the world.^39^^,^^43^ Our results confirm the recent finding^48^ that the monotypic genus *Reliquia,* endemic to a single mountain range in tropical Colombia is nested in the mostly Holarctic genus *Pontia* (including *Baltia*). *Pontia* inhabits colder environs at similarly high elevations in the northern hemisphere. This observation partially uncovers the intriguing interplay of Pieridae biogeography and adaptation to high elevations around the world. Furthermore, our BAMM analysis highlighted heightened speciation rates in some temperate pierid clades.

### Phylogenetic relationships of Pieridae

Our study provides a robust phylogenetic framework for pierid butterflies and a foundation for testing many macroevolutionary questions. We show, with strong support (UFBoot and SH-aLRT = 100), that Dismorphiinae are sister to a clade containing Coliadinae, Pseudopontiinae, and Pierinae, in agreement with Wahlberg et al.^44^ and Kawahara et al*.*^25^ All tribal relationships of Pierinae were strongly supported and differ from previous studies.^25^^,^^44^^,^^50^ All of the Pieridae sequence data used by Kawahara et al.^25^ are incorporated into our dataset, but the inferred trees differ in tribal topology (Figures 2 and S1). This is likely a result of our considerably increased taxon sampling.

We recovered the subfamily Pseudopontiinae on an extremely long branch (as shown previously in the study of Mitter et al.^63^) of approximately 50 My in length (Table S1), which likely contributes to the difficulty of confidently inferring the phylogenetic placement of this subfamily. Topological uncertainty hinders inference of ancestral states of the group as well. The morphologically unique Afrotropical subfamily Pseudopontiinae was thought to only include a single species until 2011,^43^^,^^63^ when molecular data demonstrated the existence of four additional cryptic species. Even though we incorporated additional molecular data for several species in the group, support for its position in relation to the other subfamilies is still low (as in Espeland et al.^49^ and Chazot et al.^50^). We also find that several genera, including *Euchloe* and *Ganyra*, are not monophyletic (Figure 1), and will require further taxonomic investigations.

The Pieridae crown age of around 70 Ma overlaps the K-Pg (Cretaceous–Paleogene) boundary, a major extinction event. The lineage giving rise to the family pre-dated this event, and pierids started diversifying during the Paleogene (Figure S2).

### Limitations of the study

Divergence time estimation for butterflies has been historically difficult due to the notable underrepresentation of butterflies in the fossil record. We included an analysis with the most rigorously justified relevant butterfly fossils from recent works as well as a set of analyses using secondary calibrations but consider that the use of secondary calibrations themselves is a limitation on our downstream analyses. Future discovery of butterfly fossils will ideally be needed to help solidify our understanding of butterfly divergence times. Further, some of the analyses we conducted do not allow the fraction of missing species to be specified (the sampling fraction), and thus our ∼50% sampling of Pieridae could have biased some results.

## STAR★Methods

### Key resources table

**Table:** 

REAGENT or RESOURCE	SOURCE	IDENTIFIER
**Biological samples**		

Lepidoptera tissue samples	This paper	https://doi.org/10.5061/dryad.c59zw3rbh

**Deposited data**		

Alignments used for phylogenetic analyses	This paper	https://doi.org/10.5061/dryad.c59zw3rbh
Trees produced from all analyses	This paper	https://doi.org/10.5061/dryad.c59zw3rbh
Published sequence data	Various	https://doi.org/10.5061/dryad.c59zw3rbh

**Software and algorithms**		

GeneDumper v.0.8	This paper	https://github.com/sunray1/genedumper
MAFFT v.7.0.1	Katoh and Standley^64^	https://mafft.cbrc.jp/alignment/software/
AliView v.1.27	Larsson^65^	https://ormbunkar.se/aliview/
FASconCAT-G v.1.0.4	Kück and Meusemann^66^	https://bonn.leibniz-lib.de/en/research/research-centres-andgroups/fasconcat-g
IQ-TREE v.2.0.3	Minh et al.^67^; Nguyen et al.^68^	http://www.iqtree.org
TreePL	Smith and O'Meara^69^	https://github.com/blackrim/treePL
RAxML v.8.2.12	Stamatakis^70^	https://github.com/amkozlov/raxml-ng
TreeAnnotator v.1.10.4	Drummond et al.^71^	https://beast.community/treeannotator
occCite v.0. 4.9	Owens et al.^72^	https://cran.r-project.org/web/packages/occCite/index.html
rgbif	Chamberlain et al.^73^	https://cran.r-project.org/web/packages/rgbif/index.html
ggplot2 v.3.3.5	Wickham^74^	https://ggplot2.tidyverse.org/
raster v.3.4–10	Hijmans^75^	https://cran.r-project.org/web/packages/raster/index.html
BAMMtools v.2.1.6	Rabosky et al.^76^	http://bamm-project.org/
RevBayes	Höhna et al.^77^	https://revbayes.github.io/
castor v.1.5.7	Louca and Doebeli^78^	https://cran.r-project.org/web/packages/castor/index.html
nlme v.3.1–149	Pinheiro and Bates^79^	https://cran.r-project.org/web/packages/nlme/index.html
diversitree v.0.9–15	FitzJohn^80^	https://cran.r-project.org/web/packages/diversitree/index.html
Trim Galore! V.0.40	Krueger^81^	https://www.bioinformatics.babraham.ac.uk/projects/trim_galore/

### Resource availability

#### Lead contact

Further information and requests should be directed to the lead contact, Ana P. S. Carvalho (apsdecarvalho@gmail.com).

#### Materials availability

This study did not generate new unique reagents.

#### Data and code availability

• Nucleotide, sequencing, and associated datasets have been deposited on GenBank and Dryad and are publicly available as of the date of publication. DOIs are listed in the key resources table.
• All original code has been deposited on Dryad and is publicly available as of the date of publication. DOIs are listed in the key resources table.
• Any additional information required to reanalyze the data reported in this paper is available from the lead contact upon request.

### Method details

#### Molecular data

##### Locus sampling

We obtained sequence data in 3 different ways. These were: 1) the anchored hybrid enrichment (AHE) BUTTERFLY2.0 kit,^82^ which targets 13 genes, 2) AHE BUTTERFLY1.0 ^49^, which targets 425 genes, and 3) publicly available sequence data. Details of these approaches are outlined below.

##### Anchored hybrid enrichment

We sequenced DNA from 338 Pieridae species and two outgroups sourced from various alcoholic and dry insect collections (see full sample list on Dryad at https://doi.org/10.5061/dryad.c59zw3rbh) using methods described in Kawahara et al*.*^25^ The BUTTERFLY2.0 AHE probe set captured up to 13 loci commonly used in Sanger sequencing studies of past decades.^82^^,^^83^

Raw sequencing reads underwent filtering and quality checks for base call quality (phred >20) and length (>30 bases) using Trim Galore! v.0.40^81^; and assembled via a custom Python v.2.7.6 script that performs an iterative baited assembly (IBA)^84^ for each locus. IBA uses similarity searching in usearch^85^ to assemble loci beginning with a database of reference loci from a closely related species (in this case *Danaus plexippus*) using bridger.^86^ Second, assembled loci were filtered by blasting the original probe regions of the assembly against the *Danaus plexippus* reference genome^87^ followed by mapping single hits to check for orthology. We screened the orthologous loci for sequence contamination and removed any sequences with 99% similarity to species in another family. The resulting sequences were aligned with MAFFT v.7.0.1.^64^ Different copies of DNA were collapsed into consensus sequences using FASconCAT-G v.1.0.4.^66^

We augmented the dataset with 103 pierid species sequenced by Kawahara et al.^25^ with the BUTTERFLY1.0 probe set^49^ which have up to 425 AHE loci (including the same 13 loci in BUTTERFLY2.0).

##### Published sequences

We complemented the AHE molecular dataset with sequences of up to 13 loci (the same captured in BUTTERFLY2.0) from 134 pierid species in GenBank downloaded using the software GeneDumper v.0.8 (https://github.com/sunray1/genedumper).

We used the 13 loci captured by BUTTERFLY2.0 from *Colias erate* as a reference sequence for BLAST to find other available Pieridae sequences in GenBank. GeneDumper can integrate taxonomic name updates in the GenBank search and thus requires a synonymic list to determine name validity. We used the species list of Lamas.^34^ After the original BLAST, GeneDumper filters and checks the sequence hits. When multiple sequences for the same locus of a species are available, GeneDumper considers the characteristics of the data and selects the longest sequence with the most unambiguous DNA content. The identification of the selected sequences are verified via a self-blast against GenBank’s nucleotide collection. A tiling approach is also used, where sequences are tiled along the bait sequence used in the original BLAST and the least number of tiles providing the highest coverage across the baits are chosen. Therefore, one species may be represented by sequences from multiple specimens if there are no sequences available that fully cover the bait sequence (Further details of the method can be found at https://github.com/sunray1/genedumper).

The sequences of Warren-Gash et al.^47^ were made available on GenBank after we used GeneDumper, so we downloaded those sequences from the database for species not already in our molecular dataset. We also incorporated sequences from other targeted studies,^46^^,^^49^ and used genomes and transcriptomes assembled to the BUTTERFLY1.0 probe kit in Kawahara et al.^25^ (including two as outgroups).

There were 15 species for which we created chimeras by adding cytochrome oxidase subunit 1 (COI) from one sample to another with more loci available but no COI (see full sample list on Dryad at https://doi.org/10.5061/dryad.c59zw3rbh).

##### Combining molecular datasets

All genetic sequences were then combined to create the molecular data matrix. This method of creating datasets where molecular coverage varies greatly has been shown to yield high-quality phylogenetic reconstructions if strategic taxon selection of species is taken into account for multilocus sequencing.^88^ We undertook this process by first combining data from the two AHE datasets. The 13 loci of BUTTERFLY2.0 are included (as loci numbers 1 through 13) in BUTTERFLY1.0^49^ and simplify concatenation and combination of datasets. We then manually added GenBank sequences to this genetic dataset, and subsequently aligned each locus with MAFFT following the gene alignment protocol of Breinholt et al*.*^84^ After alignment, each locus was examined in AliView v.1.27^65^ to check for indels and determine reading frames. Loci were concatenated with FASconCAT-G v.1.0.4 ^66^ to create a concatenated dataset of 425 loci.

The final dataset had fifteen outgroups with at least two species from each of the six other butterfly families. The taxonomy of butterfly names follows Lamas.^34^ In total, 593 pierid species and 15 outgroups are represented in our final concatenated dataset with up to 425 loci and 180,381 base pairs per species. Missing data is variable due to the inclusion of taxa limited to 13 loci and to 425 loci, see Dryad for a complete breakdown of loci recovered on a per-species basis.

##### Partitioning, tree inference, and branch support

The best nucleotide partition schemes were identified using ModelFinder as implemented in IQ-TREE v.2.0.3.^67^^,^^68^ For this analysis, we allowed partitions to be identified and merged with '-m TESTNEWMERGEONLY' to limit overparameterization. To reduce computation times, we used the rcluster method (set to 10, with a maximum of 1000) and limited the model set to GTR. We consolidated the original 425 locus partitions into 69 partitions but did not further partition these by codon positions to avoid extremely high numbers of *a priori* partitions for such a large taxon set. Separately, we ran ModelFinder to identify the best models of nucleotide evolution for our partitions using all available models. With these 69 partitions and models, we then performed 100 independent IQ-TREE tree searches with 1000 Ultrafast bootstraps (UFBoot) and 1000 Shimodaira-Hasegawa approximate likelihood ratio test (SH-aLRT) replicates to test for branch support. We implemented the '-bnni' command when running these analyses to alleviate concerns about model violation inherent in the Ultrafast bootstrap method.^89^ All phylogenetic analyses were run on the University of Florida HiPerGator2 Cluster (https://www.rc.ufl.edu/about/hipergator/).

##### Divergence time estimation

We used two different secondary calibration schemes obtained from the trees of Espeland et al.^49^ and Kawahara et al.^53^ to estimate pierid divergence times (Table S4). Ages, based on 95% confidence intervals in these studies, were treated as hard minimum/maximum ages and we employed the penalized likelihood tree dating with TreePL.^69^ TreePL requires an input tree, for which we used the most likely tree (highest likelihood of 100 runs) inferred with IQ-TREE. TreePL outputs divergence times as single dates per node. In order to provide a range of dates for each divergence event, we performed TreePL independently 100 times and summarized results, following the approach of St Laurent et al*.*^90^ This method uses several custom Python scripts (available at https://github.com/sunray1/treepl) and creates 100 bootstrap replicates in RAxML^70^ v. 8.2.12 using the 425-locus data matrix, partitioned according to the same 69 partitions inferred with ModelFinder. These 100 bootstrap alignments were then used to infer branch lengths on the constrained input phylogeny. We generated 100 bootstrap trees, utilizing optimization parameters that were determined with the 'prime' command and random subsample and replicate cross-validation with the 'randomcv' command for identifying the best smoothing parameter. This optimization and smoothing step was performed three times on each tree to identify the best combination of parameters and assess consistency in the smoothing parameter. All analyses used the 'thorough' command in TreePL. These analyses resulted in 100 dated trees varying only in branch lengths, which were summarized as a maximum clade credibility (MCC) tree in TreeAnnotator v.1.10.4 in the BEAST package.^71^ The MCC tree was used in all downstream analyses that required a dated phylogeny.

In addition to making use of secondary calibrations in TreePL, we conducted an analysis using fossil calibrations as the minimum ages. To calibrate the root, a median age of Angiosperms (139.4 Ma) from Magallón et al.^91^ was used (as done by Espeland et al.^49^). In total, eight fossil calibrations were applied to the following places in the tree (fossil names and their age noted in parentheses): 1) the Parnassiinae stem age (*Thaites ruminiana*, 23 Ma); 2) the Hesperiidae stem (*Protocoeliades kristenseni*, 54 Ma); 3) an internal Hesperiidae crown age (*Pamphilites abdita*, 23 Ma); 4) Nymphidiini (Riodinidae) stem (*Theope* sp., 15 Ma); 5) the crown node of Satyrinae + Heliconiinae (Nymphalidae) (*Vanessa amerindica*, 33.7 Ma); 6) *Aporia* (Pieridae) crown age (*Aporia* cf. *crataegi*, 2.6 Ma); 7) *Belenois* (Pieridae) crown age (*Belenois crawshayi*, 0.02 Ma); 8) split between Coliadinae and (Pseudopontiinae + Pierinae) (*Vanessa pluto*, 16 Ma). Each of these fossils were selected because they can be confidently identified as belonging to a particular lineage, making them well-suited to calibrate dating analyses.^92^ TreePL was run on this dataset as noted above, with the exception that the root was a hard maximum age and all fossils were hard minimum ages. For our discussion, we focus on estimated dates obtained using calibrations from Kawahara et al.*,*^53^ because this is the most comprehensive, fossil-calibrated dated phylogeny of Lepidoptera to date.

#### Distribution and bioclimatic data

We used the R library occCite v.0.4.9^72^ to assemble distribution data. It provides a platform to compile georeferenced data from global databases while also generating a list of primary data sources for each record from institutional collections. We extracted occurrence data using the command 'occQuery' and followed the taxonomy of Pieridae according to Lamas.^34^ This command checks species names against the Global Names Index (gni.globalnames.org), then uses rgbif^73^ to send a query to GBIF,^93^ requesting all records with geographic coordinates. We removed records that did not have decimal places to increase accuracy and removed records with identical coordinates to the first four decimal places because they were too precise to provide meaningful additional information. Original occurrence data sources with digital object identifiers (DOIs) were gathered using 'occCitation'. We also extracted additional occurrence data from the literature and directly from GBIF for 37 species that were not obtained using the method described above (see full reference list on Dryad at https://doi.org/10.5061/dryad.c59zw3rbh).

We mapped all records by species in an atlas using the package ggplot2 v.3.3.5,^74^ with a custom R script (https://github.com/hannahlowens/PieridaeDiversity). The atlas was inspected for occurrence points that appeared as outliers or errors based on published distribution maps and opinions of Pieridae experts (authors and collaborators - see Acknowledgments) and we subsequently removed these putatively erroneous records. We also combined the records of the cryptic species *Leptidea sinapis*, *L. juvenica* and *L. reali* as the “sinapis complex” due to difficulty in delimiting distribution in these species.^94^ The final dataset had over 800,000 records for 541 pierid species, representing 91% of species in the phylogeny (https://doi.org/10.5061/dryad.c59zw3rbh). We wanted to have present day representation of distribution for a more informative dataset on abiotic requirements, therefore we did not remove records that could be associated with established introductions and migration.

We extracted bioclimatic data for the geographic locations of these records from the WorldClim dataset (2.5 arc-minute resolution^95^) using the package raster v.3.4–10.^75^ Since climatic stability can refer to different timescales including paleoclimates,^96^ we use the phrase "temperature stability" to refer to annual variability (seasonality) and daily variability, depending on the context. Considering our hypotheses regarding the roles of extreme conditions and seasonality, we focused on the following variables: annual mean temperature (average of BIO1), mean diurnal temperature range (average of BIO2, which is the mean of (daily max temp - daily min temp) within a month), maximum temperature in the warmest month (95% quantile of BIO5), minimum temperature in the coldest month (5% quantile BIO6), and temperature annual range (average of BIO7). The averages (BIO1, BIO2, BIO7), or 5% (BIO6) or 95% (BIO5) quantiles were calculated for each species that had at least one record. The summarized data can be found on Dryad (https://doi.org/10.5061/dryad.c59zw3rbh).

A maximum likelihood-based ancestral state reconstruction (ASR) was conducted in Phytools using the 'contMap' function ('anc.ML' model) of all temperature-related variables using the dated Pieridae tree without outgroups.

#### Diversification and correlation analyses

We conducted a BAMM (Bayesian Analysis of Macroevolutionary Mixtures) analysis to infer diversification rates on the pierid phylogeny. The input topology was the TreePL tree, dated with secondary calibrations from Kawahara et al.*,*^53^ excluding outgroups. We estimated prior parameters using the R package BAMMtools v.2.1.6^76^ with the command ‘setBAMMpriors’ (lambdaInitPrior ∼3.33; lambdaShiftPrior ∼0.01; muInitPrior ∼3.33). We ran a reversible-jump MCMC for 100 million generations, sampling every 100,000 generations with six different expected shifts (5, 10, 15, 20, 25, and 30). We used a fraction file to account for taxon sampling bias across the tree (https://doi.org/10.5061/dryad.c59zw3rbh). BAMM output files were analyzed in BAMMtools with a 10% burn-in. We plotted shift posterior probabilities for all six analyses and determined that they converged around 20 shifts (Figure S12). Thus, the result based on 20 shifts was used for diversity analyses described below.

We also conducted a lineage-specific birth-death-shift (LSBDS) analysis in RevBayes^77^ with an MCMC chain of 10,000 steps; following the parameters employed in Höhna et al*.*^97^ Rate variables were set to lognormal distributions for both mean speciation and extinction rates, the rate of rate-shift events was set to uniform, and six rate categories were set as a global parameter. LSBDS does not have an option for fine level sampling bias, and therefore a global sampling fraction of 51% of 1,159 described species of Pieridae was applied.

Considering recent criticisms of estimating diversification rates using phylogenies of extant taxa (e.g., Louca and Pennell^98^), we also used the castor v.1.5.7 R package,^78^ to estimate pulled speciation rates (PSR) and deterministic lineage through time plots (dLTT). To ensure time slices are biased more toward the present where extant time trees are expected to be more reliable, we first used the function 'fit_hbd_psr_on_grid' with arguments 'Ngrid' set to 15, and 'Ntrials' to 100 in order to get density values for X and Y axes. These density values were used as arguments in the castor function 'castor::get_inhomogeneous_grid_1D' to create an inhomogeneous 'age_grid' which could then be used as an argument in the function 'fit_hbd_psr_on_grid' to calculate pulled speciation rate (PSR) and dLTTs on the inhomogeneous grid.

To assess a possible correlation between temperature and rates of diversification, we calculated phylogenetic generalized least squares (PGLS) in the R package nlme v.3.1–149.^79^ Speciation and extinction rates were extracted from the results from the BAMM analysis using the 'getTipRates' command in BAMMtools for all species in the phylogeny. With the command 'gls', we set the correlation function to Brownian and method to 'ML'. We performed ANOVA tests on resultant PGLS models to check for significance and identify the relationship of diversification rates and variable (slopes) as positive or negative.

As another means of testing for the relationship between temperature and speciation rates, we used Quantitative State Speciation and Extinction (QuaSSE) as implemented in diversitree v.0.9–15.^80^ We used a pruned tree including only species with available trait data (541 species). Our methods largely followed Zhang et al.*,*^99^ whereby we tested increasingly complex QuaSSE models to create likelihood functions where speciation is constant, linear, sigmoidal, or hump-shaped, with and without a drift parameter. The Akaike information criterion (AIC) was used to identify the best fit QuaSSE model for each of the BIO variables.

Climatic BIO variables used in the analysis above stem from present day temperatures. To evaluate how global temperature changes through geological time affected diversity in the family as well as its most diverse subfamily Pierinae, we fitted four temperature-dependent models to our phylogeny in the R. We also considered the possibility of time influencing diversity by fitting six time-dependent models. To run these analyses, we used the pipeline and custom R scripts developed by Condamine et al*.*^100^ Specifically, time-dependent models were derived from Morlon et al.^101^ and Stadler^102^ and the paleoclimate models from Condamine et al*.*^103^ The original script provided by the authors included linear and exponential models, but due to criticism directed toward linear diversification dependency models by Gamisch^104^ (but also see Morlon et al.^105^), we commented out code for linear models. The time-dependent models were fitted with maximum likelihood using the ‘fit_bd’ function and the paleoclimate models used the ‘fit_env’ function, both functions available in the pipeline of Condamine et al*.*^100^ Sampling fractions of 0.5 for Pieridae and 0.44 for Pierinae were assigned to account for the proportion of taxon coverage in the phylogenies.
