# Supplementary material for: Comprehensive phylogeny of Pieridae butterflies reveals strong correlation between diversification and temperature
Source: iScience. 2024 Feb 28;27(4):109336. doi: 10.1016/j.isci.2024.109336 (PMC10945170; doi:10.1016/j.isci.2024.109336)
Supplement: Document S1. Figures S1–S12 and Tables S1–S4 [file mmc1.pdf]

iScience, Volume *27*

Supplemental information

Comprehensive phylogeny of Pieridae butterflies reveals strong correlation between diversification and temperature

Ana Paula S. Carvalho, Hannah L. Owens, Ryan A. St Laurent, Chandra Earl, Kelly M. Dexter, Rebeccah L. Messcher, Keith R. Willmott, Kwaku Aduse-Poku, Steve C. Collins, Nicholas T. Homziak, Sugihiko Hoshizaki, Yu-Feng Hsu, Athulya G. Kizhakke, Krushnamegh Kunte, Dino J. Martins, Nicolás O. Mega, Sadaharu Morinaka, Djunijanti Peggie, Helena P. Romanowski, Szabolcs Sáfián, Roger Vila, Houshuai Wang, Michael F. Braby, Marianne Espeland, Jesse W. Breinholt, Naomi E. Pierce, Akito Y. Kawahara, and David J. Lohman

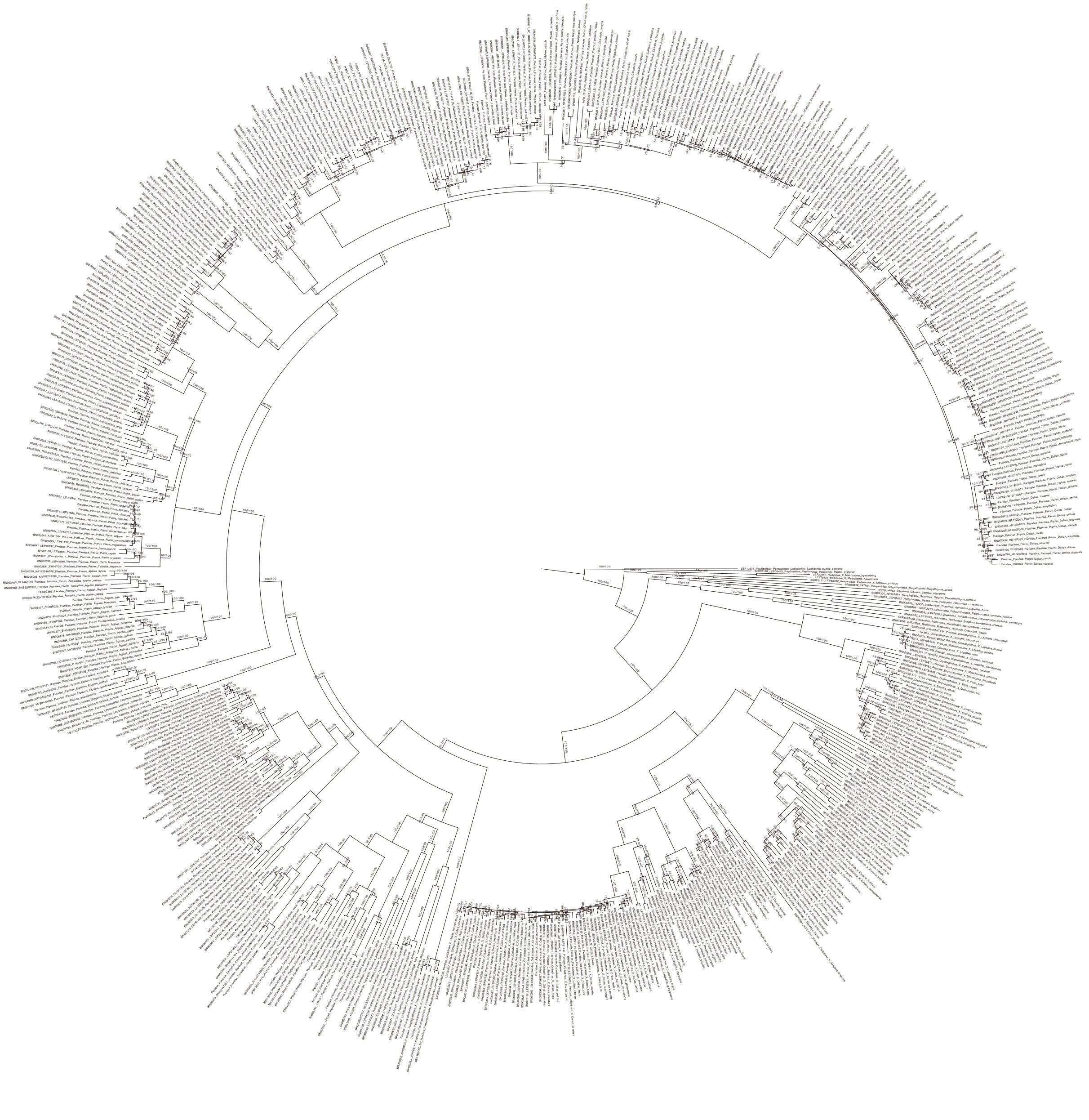

Fig. S1. The best of 100 IQ-TREE analyses based on a concatenated supermatrix of 425 AHE loci, partitioned according to ModelFinder. Support values are UFBoot/SH-aLRT, this tree was used as the input topology for dating in TreePL and is related to Figure 1.

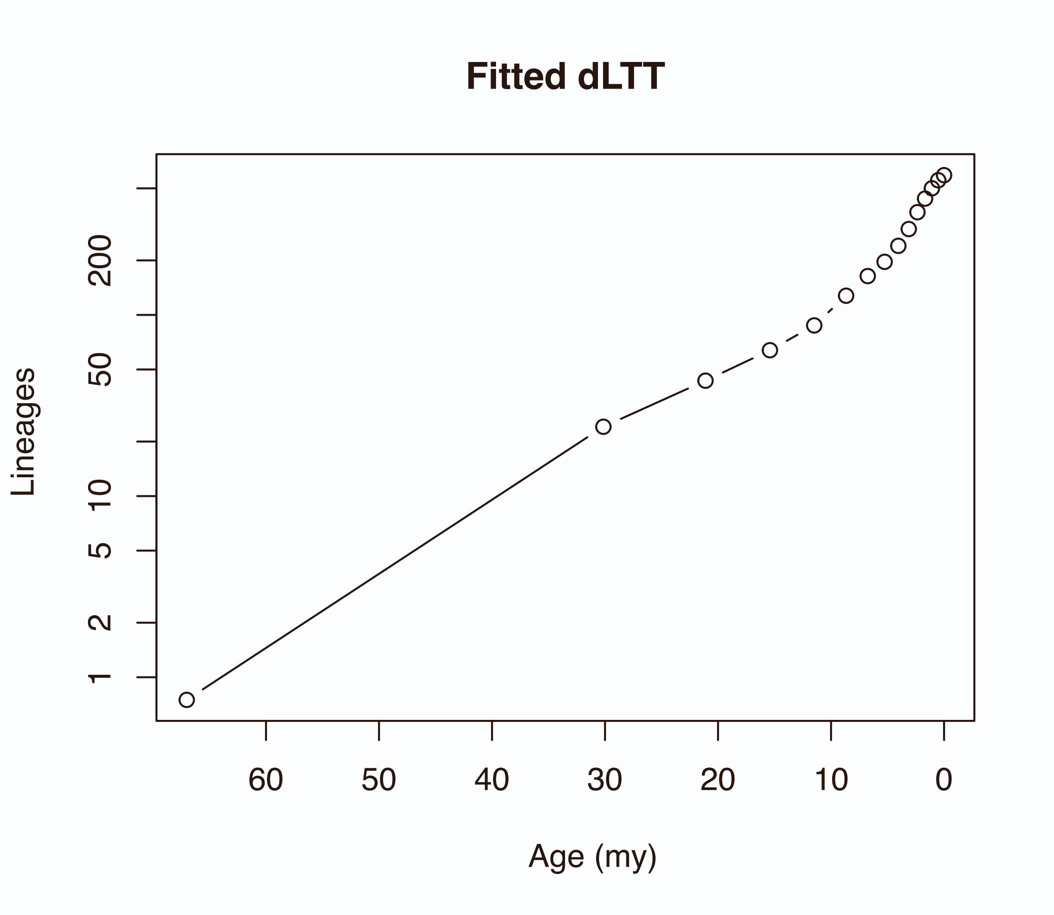

Fig. S2. Fitted deterministic Lineage Through Time (dLTT) plot of Pieridae conducted in castor on an inhomogenous time grid of 15 time slices, related to STAR method castor.

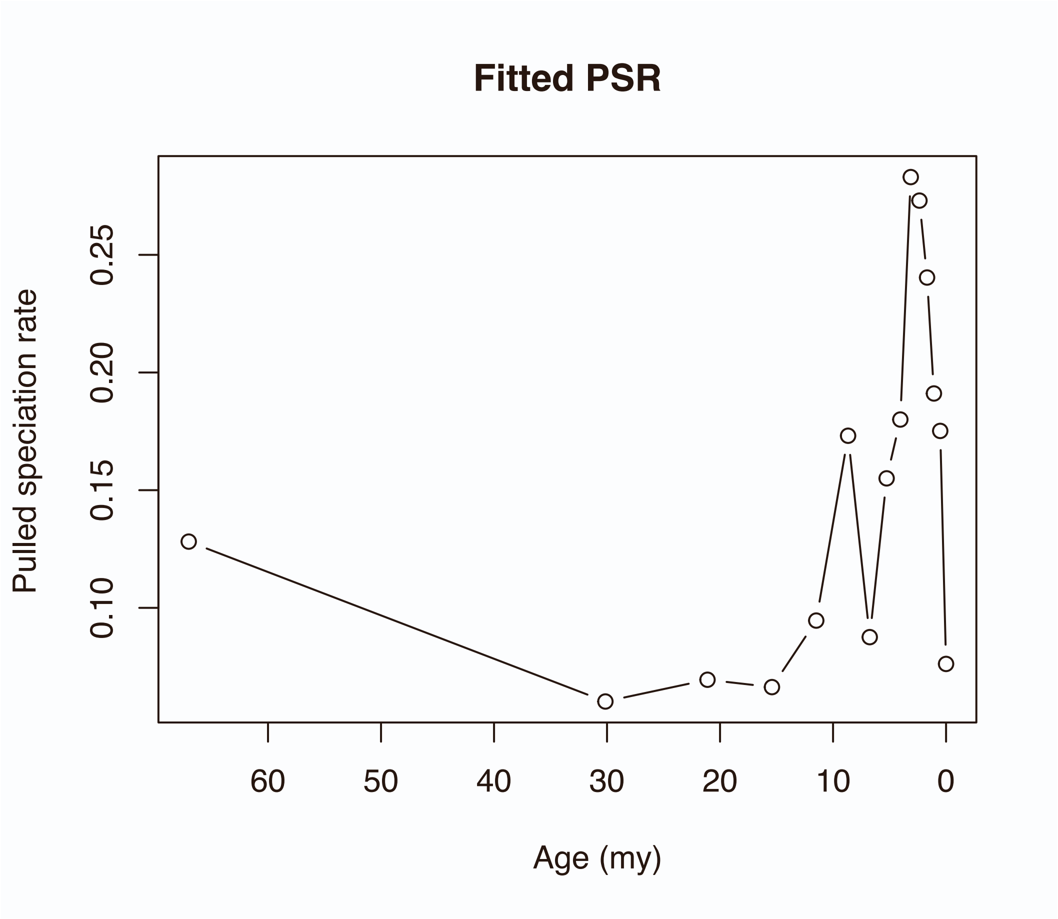

Fig. S3. Fitted Pulled Speciation Rate (PSR) plot of Pieridae conducted in castor on an inhomogenous time grid of 15 time slices, related to STAR method castor.

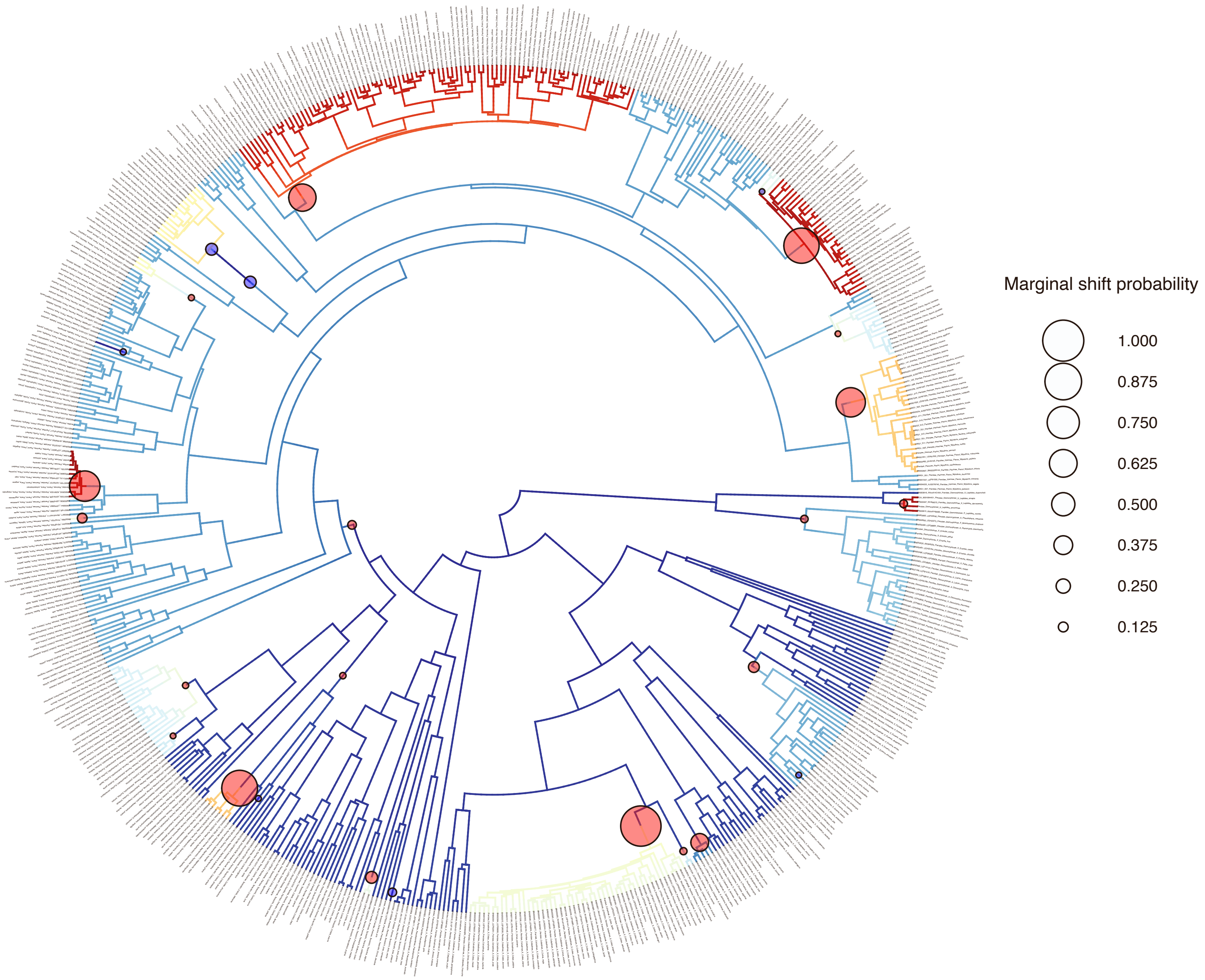

Fig. S4. STAR method BAMM's net diversification rates Distinct Shift Configuration, related to Figure 1.

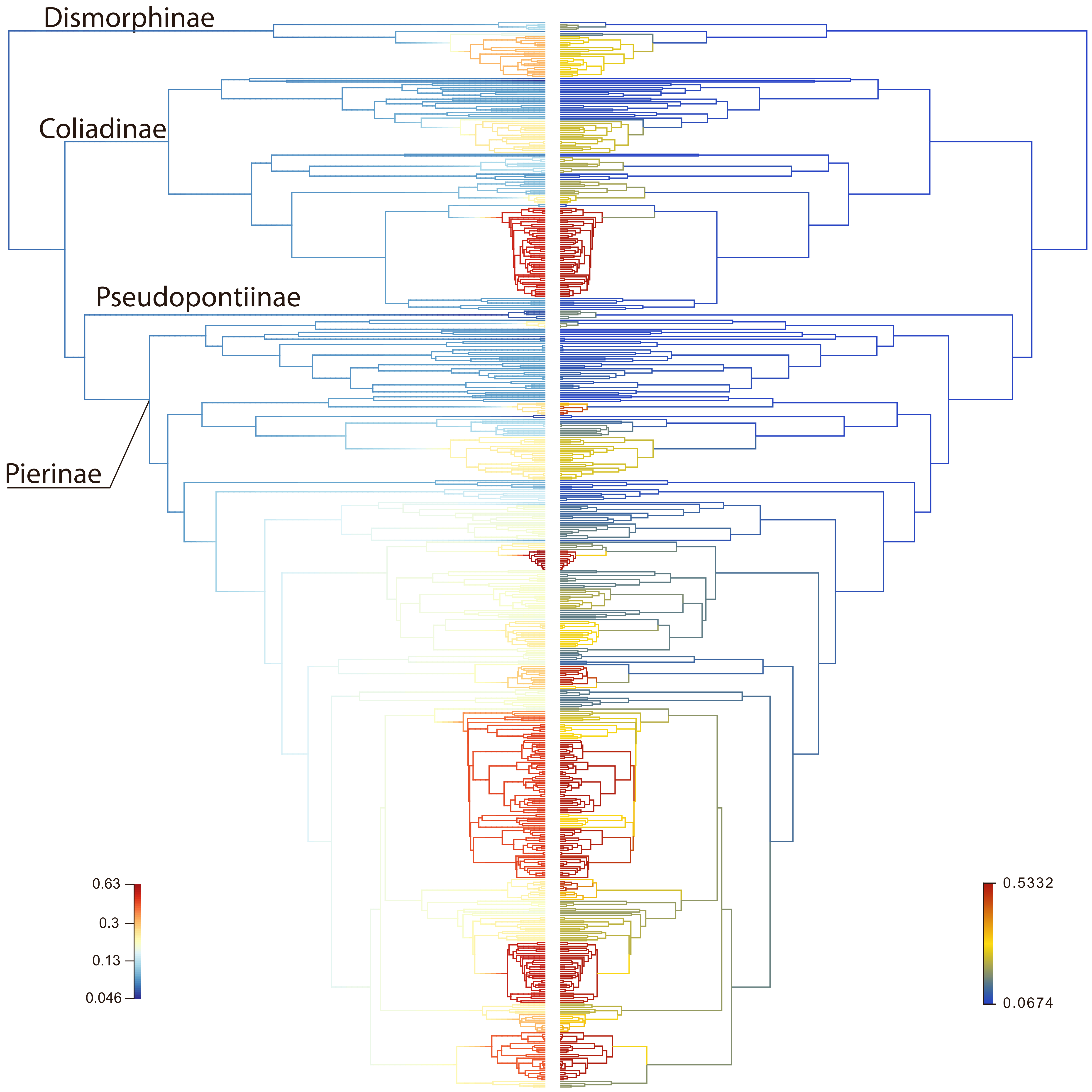

Fig. S5. Diversification rate estimations from lineage-specific birth-death shift analyses in STAR method BAMM (left) and STAR method RevBayes (right), BAMM rates are related to Figure 1.

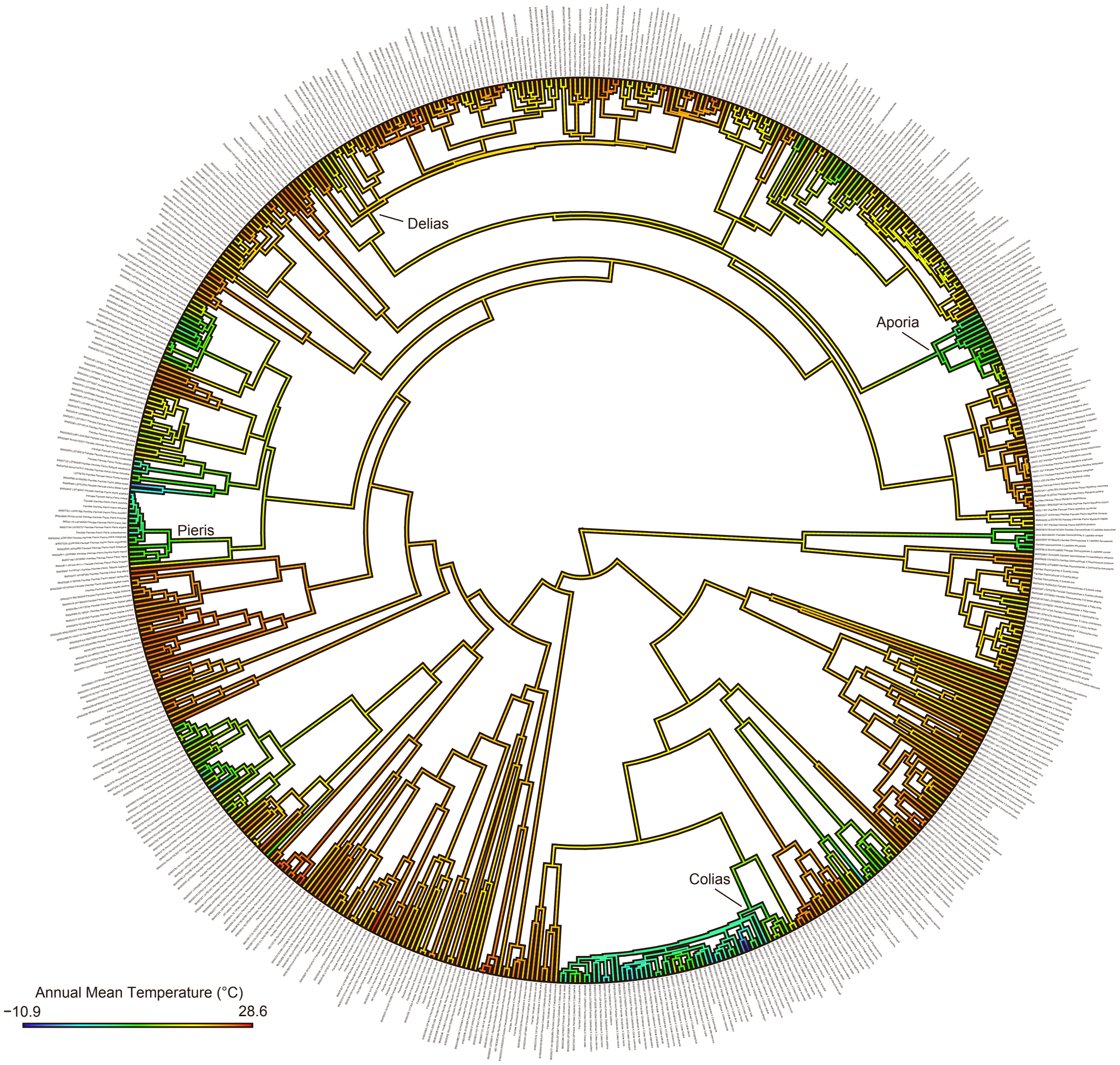

Fig. S6. Ancestral state reconstruction of annual mean temperature (BIO1, WorldClim) conducted with contMap, related to STAR method Phytools.

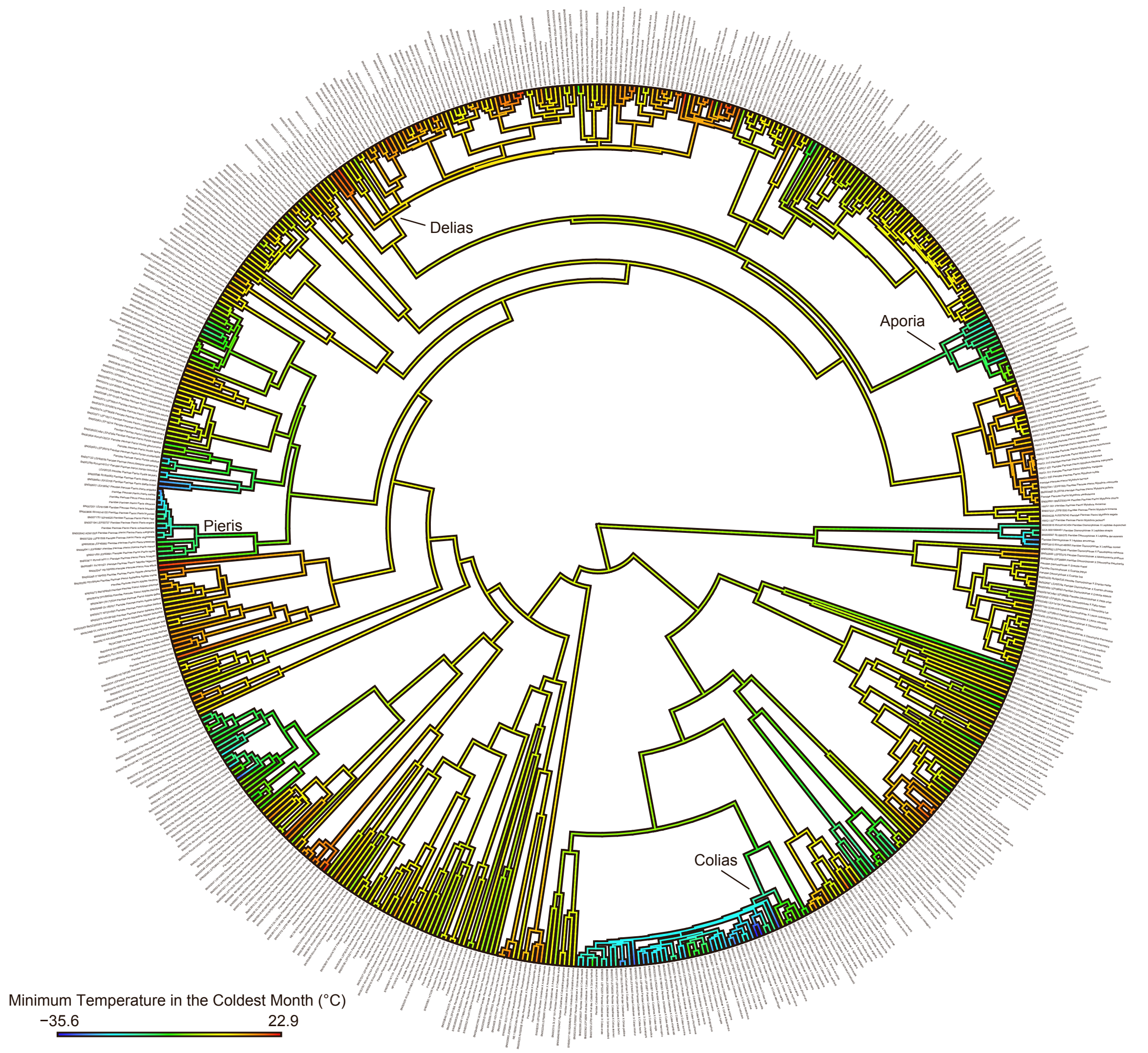

Fig. S7. Ancestral state reconstruction of minimum temperature in the coldest month (BIO6, WorldClim) conducted with contMap, related to STAR method Phytools.

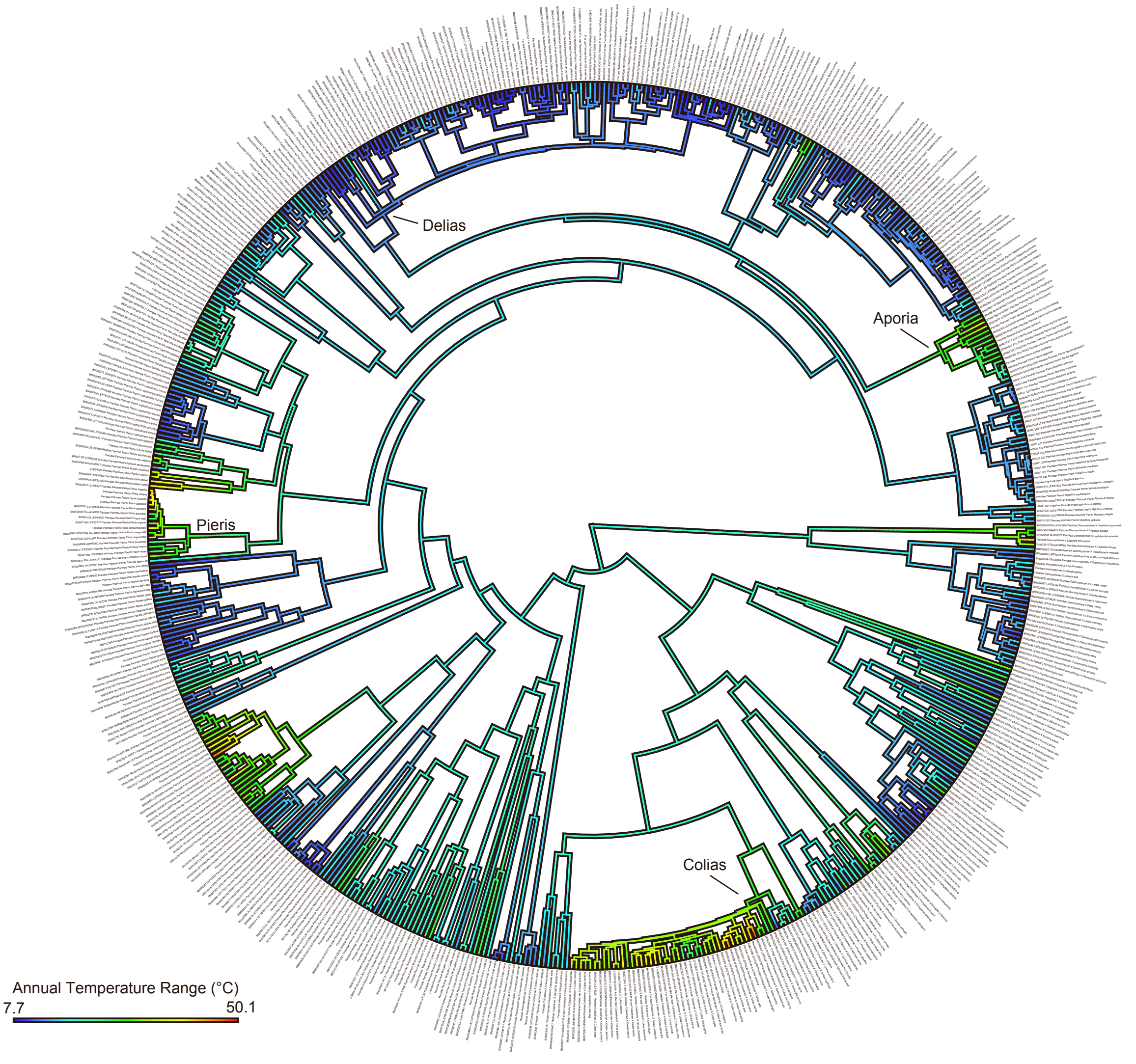

Fig. S8. Ancestral state reconstruction of temperature annual range (BIO7, WorldClim) conducted with contMap, related to STAR method Phytools.

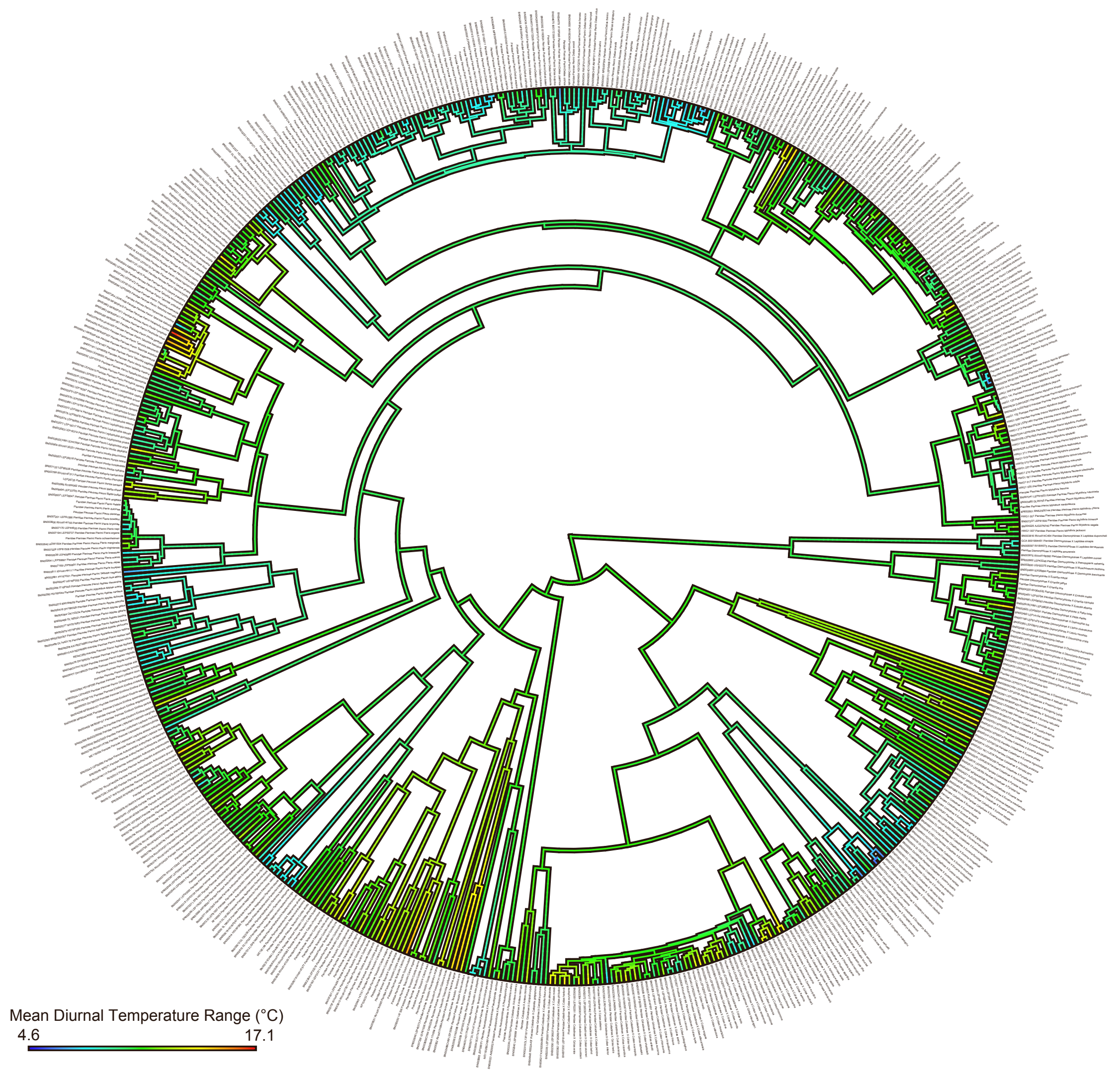

Fig. S9. Ancestral state reconstruction of annual mean diurnal temperature range (BIO2, WorldClim) conducted with contMap, related to STAR method Phytools.

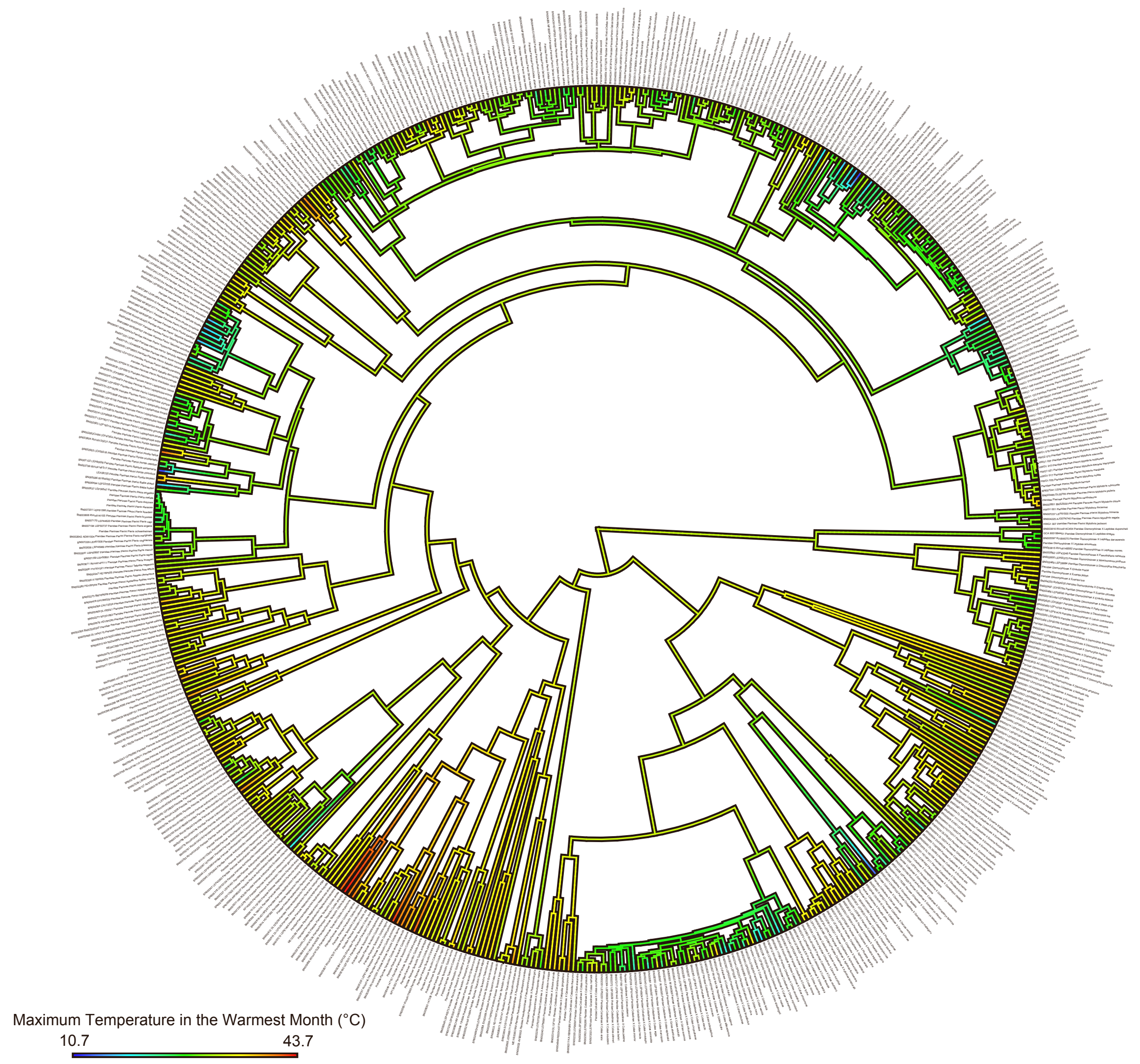

Fig. S10. Ancestral state reconstruction of maximum temperature of the warmest month (BIO5, WorldClim) conducted with contMap, related to STAR method Phytools.

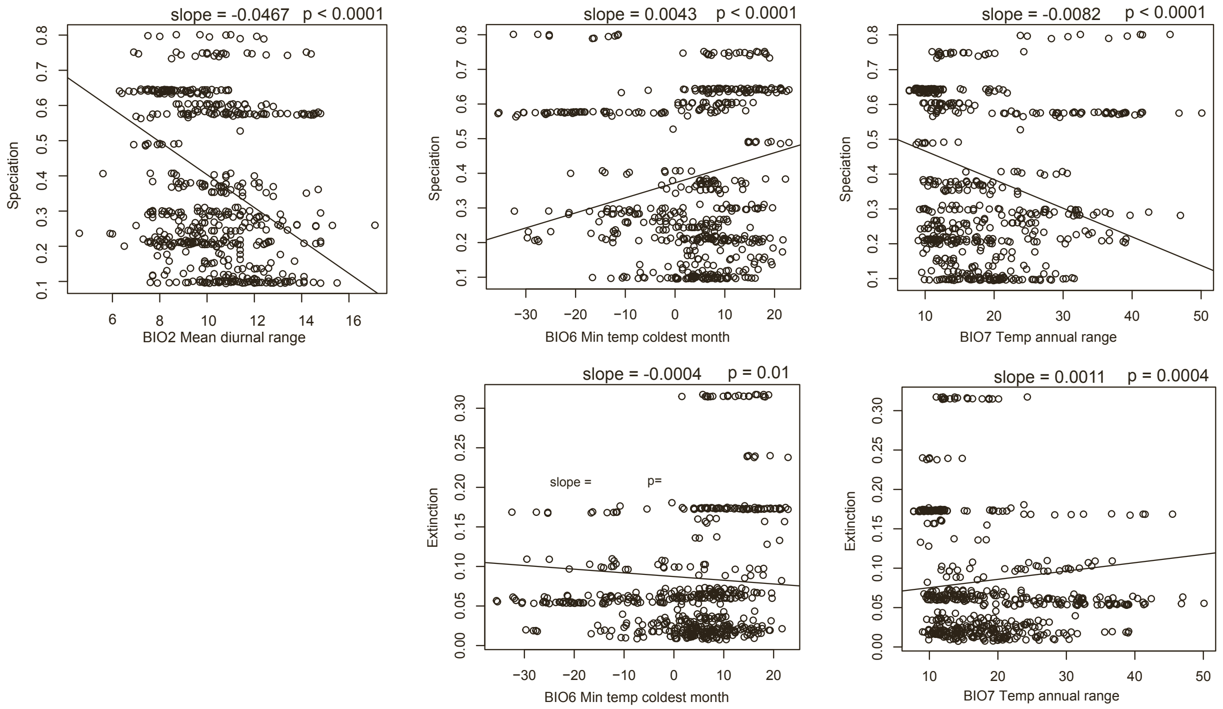

Fig. S11. Phylogenetic generalized least squares (PGLS) analysis comparing speciation and extinction rates (estimated by STAR method BAMM) with WorldClim BIO variables, only significant ($p \leq 0.01$) analyses are shown; this figure is relaed to STAR methods BAMM and nlme.

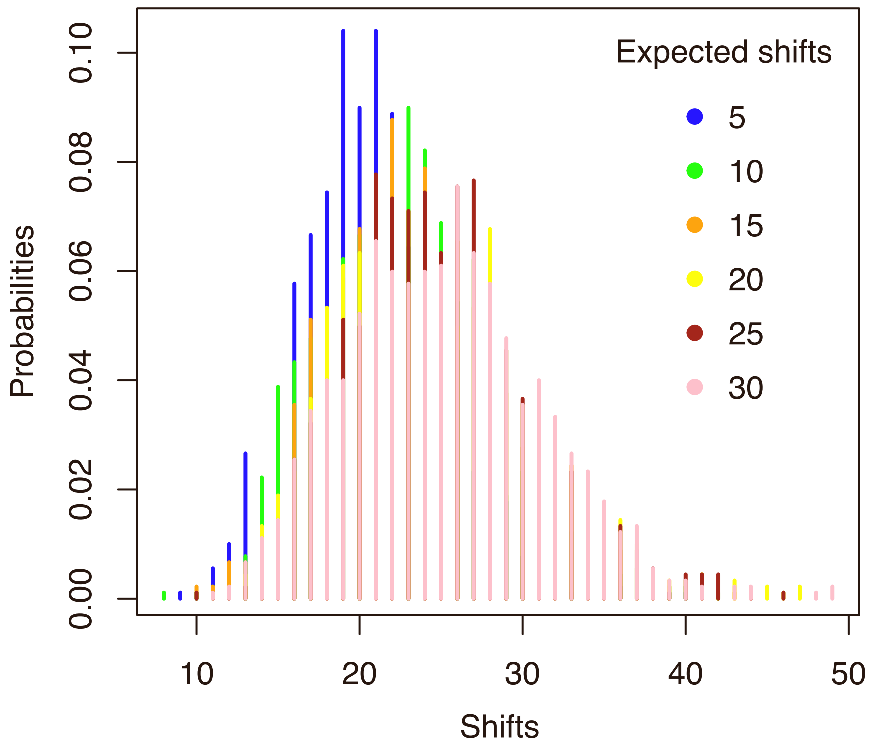

Fig. S12. BAMM posterior probability distributions with alternative expected number of diversification rate shifts, converging around 20, related to STAR method BAMM.

Table S1. Mean (minimum, maximum) dates for Pieridae from dating in TreePL and its monophyletic lower taxa under three schemes used to date the tree, these dates are related to Figure 1 and STAR method TreePL.

Node	Calibration scheme: Kawahara et al. 2019		Espeland et al. 2018		All fossils + Papilionoidea secondary	
	**Crown**	**Stem**	**Crown**	**Stem**	**Crown**	**Stem**
Pieridae	67.0149 [64.5, 69.6]	79.6154 [76.1, 83.5]	90.6196 [84.5, 98.1]	107.2195 [99.1, 118.1]	81.0413 [78.6, 85.7]	93.5524 [92.1, 97.7]
Dismorphiinae	33.9027 [32.7, 35.0]	67.0149 [64.5, 69.6]	44.6404 [42.1, 46.8]	90.6196 [84.5, 98.1]	41.4309 [38.1, 44.8]	81.0413 [78.6, 85.7]
Coliadinae	47.0301 [44.2, 49.8]	60.0086 [58.1, 61.9]	62.2908 [57.4, 66.0]	81.5742 [76.2, 87.7]	57.7456 [54.9, 68.2]	73.6964 [70.8, 79.8]
Pseudopontiinae	4.5295 [3.8, 5.1]	57.5307 [55.7, 59.1]	5.8562 [5.2, 6.7]	78.3034 [73.1, 83.9]	5.5253 [4.8, 6.6]	70.9325 [67.5, 78.3]
Pierinae	49.4118 [48.9, 49.9]	57.5307 [55.7, 59.1]	67.899 [64.9, 72.2]	78.3034 [73.1, 83.9]	62.4482 [59.9, 70.6]	70.9325 [67.5, 78.3]
Teracolini	42.3856 [41.4, 43.3]	49.4118 [48.8, 49.9]	57.2919 [54.3, 60.4]	67.899 [64.9, 72.2]	53.2399 [50.5, 59.0]	62.4482 [59.9, 70.6]
Nepheroniini	27.0507 [25.6, 29.2]	42.86.08 [40.8, 44.9]	36.484 [33.8, 40.7]	58.9792 [54.4, 66.2]	33.8078 [31.5, 38.0]	54.2904 [46.0, 60.5]
Anthocharidini	36.123 [28.9, 42.2]	42.86.08 [40.8, 44.9]	49.1686 [37.1, 62.7]	58.9792 [54.4, 66.2]	45.5641 [35.5, 53.9]	54.2904 [46.0, 60.5]
Leptosianini	13.7 [11.3, 15.2]	45.1041 [44.4, 45.7]	18.0104 [15.3, 21.2]	62.5114 [59.0, 66.3]	17.1274 [13.9, 20.2]	57.5497 [55.1, 66.5]
Elodinini	12.5064 [10.9, 13.8]	41.1033 [40.2, 42.4]	16.4674 [14.5, 17.9]	56.5218 [53.5, 60.3]	15.5718 [12.7, 17.6]	52.1406 [49.5, 59.4]
Pierini	35.0046 [34.0, 36.0]	41.1033 [40.2, 42.4]	47.4924 [45.3, 50.8]	56.5218 [53.5, 60.3]	44.1455 [42.0, 51.0]	52.1406 [49.5, 59.4]

Table S2. Results of all temperature- and time-dependent models fit to the data using custom STAR method R scripts, models are sorted by ΔAICc.

Pieridae

Model type	means.Models	means.NP	means.logL	means.AICc	ΔAICc	means.Lambda	means.Alpha	means.Mu	means.Beta
Temp-dependent	BEnv.VarDEnv.Var_EXPO	4	-1665.703	3339.4751	0	0.31681266	0.21580677	0.310847	0.21694265
Temp-dependent	BCSTDEnv.Var_EXPO	3	-1670.65	3347.3409	7.865761	0.44262873	NA	0.28906	0.03322339
Temp-dependent	BEnv.VarDCST_EXPO	3	-1671.165	3348.3705	8.895401	0.4661739	-0.0570639	0.223456	NA
Temp-dependent	BEnv.Var_EXPO	2	-1673.668	3351.3554	11.88026	0.53219841	-0.2102633	NA	NA
Time-dependent	BTimeVarDTimeVar_EXPO	4	-1672.29	3352.648	13.17286	0.4431	-0.02746	0.28232	-0.02616
Time-dependent	BCSTDTimeVar_EXPO	3	-1673.551	3353.143	13.66786	0.4689	NA	0.36739	0.00384
Time-dependent	BCSTDCST	2	-1675.27	3354.561	15.08586	0.493	NA	0.4172	NA
Time-dependent	BTimeVar_EXPO	2	-1680.485	3364.99	25.51486	0.3226	-0.06208	NA	NA
Time-dependent	BTimeVarDCST_EXPO	3	-1680.485	3367.01	27.53486	0.3225	-0.06207	0	NA
Time-dependent	BCST	1	-1771.161	3544.329	204.8539	0.1989	NA	NA	NA

Pierinae

Model type	means.Models	means.NP	means.logL	means.AICc	ΔAICc	means.Lambda	means.Alpha	means.Mu	means.Beta
Temp-dependent	BCSTDEnv.Var_EXPO	3	-1005.691	2017.4451	0	0.50889452	NA	0.23887	0.07915947
Temp-dependent	BEnv.VarDCST_EXPO	3	-1006.278	2018.6186	1.1735	0.60664237	-0.122421	0.142719	NA
Temp-dependent	BEnv.VarDEnv.Var_EXPO	4	-1005.462	2019.0293	1.584172	0.54552491	-0.0823228	0.125484	0.06730902
Time-dependent	BTimeVar_EXPO	2	-1007.652	2019.335	1.889909	0.4169	-0.07687	NA	NA
Temp-dependent	BEnv.Var_EXPO	2	-1007.888	2019.8071	2.361986	0.65407083	-0.216513	NA	NA
Time-dependent	BTimeVarDTimeVar_EXPO	4	-1006.46	2021.026	3.580909	0.4906	-0.05649	0.1738	-0.04991
Time-dependent	BTimeVarDCST_EXPO	3	-1007.652	2021.367	3.921909	0.4169	-0.07686	0	NA
Time-dependent	BCSTDTimeVar_EXPO	3	-1009.637	2025.337	7.891909	0.5638	NA	0.39771	0.00938
Time-dependent	BCSTDCST	2	-1013.206	2030.444	12.99891	0.6178	NA	0.50696	NA
Time-dependent	BCST	1	-1065.395	2132.801	115.3559	0.2634	NA	NA	NA

Table S3. Results of PGLS and QuaSSE analyses correlating speciation rates with the WorldClim variables of annual mean temperature (BIO1), mean diurnal range (BIO2), maximum temperature in warmest month (BIO5), minimum temperature in the coldest month (BIO6), and temperature annual range (BIO7), these results are related to STAR methods PGLS and QuaSSE.

	PGLS		QuaSSE		
Variable	Correlation	P-value	Correlation	Model	ΔAIC
BIO1	Positive	p=0.8	Negative	Linear	1.689
BIO2	Negative	**p<0.0001**	Negative	Drift sigmoidal	**11.358**
BIO5	Negative	p=0.08	Negative	Drift sigmoidal	**6.514**
BIO6	Positive	**p<0.0001**	Negative	Sigmoidal	1.845
BIO7	Negative	**p<0.0001**	Positive	Sigmoidal	1.714

Table S4. Secondary calibration schemes used to estimate divergence times of Pieridae using STAR method TreePL and relate to Figure 1.

	**Calibrations**	
**Node**	**Kawahara et al. 2019**	**Espeland et al. 2018**
Papilionoidea	86.85 - 110.33	91.2 - 142.5
Parnassinae + Papilioninae	43.99 - 76.19	42.5 - 74.3
Eudaminae + Hesperiinae	33.67 - 60.72	45.3 - 73.7
Hesperiidae + Hedylidae	72.17 - 96.56	80 - 129.3
Danaus + (Satyrinae + Heliconiinae)	58.98 - 80.18	66.4 - 106.6
Aphnaeinae + Polyommatinae	38.11 - 59.75	53.9 - 86
Riodinidae + Lycaenidae	55.66 - 77.67	69 - 109.8
Colias croceus + *Phoebis sennae*	18.07 - 40.05	27.7 - 59.2
Hebomoia glaucippe + *Ascia monuste*	27.77 - 47.19	40.8 - 69.1
